# Recurrent Severe Hypophosphatemia Following Intravenous Ferric Carboxymaltose Infusion: A Case Report

**DOI:** 10.7759/cureus.108750

**Published:** 2026-05-12

**Authors:** Louis A Enchill, Ogheneakpobor E Ubogun, Shantoi Williams, Hubert Gbate, Nana Ama D Amankwa

**Affiliations:** 1 Internal Medicine, Montefiore St. Luke's Cornwall Hospital, Newburgh, USA; 2 Radiology, Korle-bu Teaching Hospital, Accra, GHA; 3 Internal Medicine, Lincoln Medical Center, New York, USA

**Keywords:** drug-induced hypophosphatemia, ferric carboxy maltose, intravenous iron infusion, severe hypophosphatemia, symptomatic hypophosphatemia

## Abstract

Hypophosphatemia is an increasingly recognized complication of intravenous (IV) iron therapy, particularly with ferric carboxymaltose (FCM). We present a case of recurrent, symptomatic hypophosphatemia requiring hospitalization following IV FCM infusions, highlighting the clinical course, management challenges, and underlying mechanisms. This case highlights the importance of early recognition, risk stratification, and laboratory monitoring in patients receiving IV iron. It also highlights the importance of carefully selecting patients who truly need IV iron per standard guidelines and the appropriate selection of a specific IV iron formulation based on the patient's background characteristics.

## Introduction

Intravenous (IV) iron formulations have become increasingly utilized for the management of iron deficiency anemia, particularly in patients who are intolerant to oral formulations or when refractory to oral formulations. Ferric carboxymaltose (FCM), a newer-generation IV iron preparation, allows for rapid administration of large iron doses and has demonstrated efficacy in quickly replenishing iron stores. However, emerging evidence has identified hypophosphatemia as a significant adverse effect associated with FCM administration, mediated through fibroblast growth factor 23 (FGF23)-induced renal phosphate wasting [1-5].

We present the case of a 34-year-old woman with iron deficiency who developed severe, persistent hypophosphatemia with secondary hyperparathyroidism and symptomatic neuromuscular manifestations following FCM infusion. Despite aggressive phosphate replacement therapy, she experienced recurrent symptomatic hypophosphatemia requiring multiple hospitalizations. This case highlights the potential severity of FCM-induced hypophosphatemia, the challenges in management despite supplementation, and the importance of checking baseline phosphate levels and monitoring phosphate levels in patients receiving this formulation. Additionally, it highlights the need for clinician awareness regarding this complication and consideration of alternative iron formulations in susceptible patients.

## Case presentation

A 34-year-old female with a history of hypothyroidism, well-controlled on levothyroxine and anxiety managed with escitalopram, presented to the emergency department with peri-oral tingling, bilateral hand numbness, and persistent muscle twitching. She had previously been managed for iron deficiency, having presented to an urgent care facility with fatigue, generalized malaise, and exertional dyspnea. Laboratory investigations at that time revealed iron deficiency with ferritin of 8 ng/mL, serum iron of 40 µg/dL, total iron-binding capacity (TIBC) of 410 µg/dL, and normal hemoglobin (13.1 g/dL) and hematocrit (39%). She was initiated on intravenous FCM, receiving two 750 mg doses two weeks prior for symptomatic non-anemic iron deficiency. The rationale for selecting intravenous iron therapy remains unclear. Based on the information provided, the patient did not appear to meet established guideline criteria for intravenous iron replacement, and it is not evident why oral iron supplementation was not considered as an initial approach. Additionally, it is uncertain whether baseline serum phosphate levels were assessed before the administration of intravenous FCM.

Within two weeks, routine follow-up showed hypophosphatemia (1.2 mg/dL). Despite oral phosphate supplementation and IV phosphate replacement during outpatient visits, phosphate levels declined further. She subsequently presented to the emergency department with muscle twitching, peri-oral tingling, and bilateral hand numbness. She denied weakness, visual changes, vomiting, dyspnea, alcohol use, or other new medication use. Physical examination revealed positive Chvostek’s sign; she was otherwise hemodynamically stable and had no positive examination findings. Laboratory evaluation at presentation is demonstrated below (Table 1), showing severe hypophosphatemia among other findings.

**Table 1:** Metabolic profile at first presentation.

Parameter (Blood levels)	Result	Reference range
Sodium	142.0	135-145.0 mmol/l
Potassium	4.1	3.5-5.1 mmol/l
Chloride	106.0	98-107.0 mmol/l
Creatinine	0.7	0.7-1.2 mg/dL
Calcium	8.4	8.5-10.5 mg/dL
Magnesium	2.0	1.7-2.8 mg/dL
Phosphate	0.8	2.5-4.5 mg/dL
Alkaline phosphatase	61.0	45.0-130.0 U/L
Ferritin	942.0	10.0-150.0 ng/mL
Iron	205.0	65.0-175.0 µg/dL
Total iron binding capacity	420.0	135.0-475.0 µg/dL
Parathyroid hormone	95.1	20.0-80.0 pg/mL
25-Hydroxyvitamin D	32.2	30.0-60.0 ng/mL
Urine phosphorus: Creatinine ratio	613.0	567.0-795.0 mg/g

These findings were consistent with secondary hyperparathyroidism and renal phosphate wasting. Nephrology consultation supported the hypothesis of IV iron-induced renal phosphate loss.

During hospitalization, she received intravenous phosphate replacement (a total of 75 mmol sodium/potassium phosphate), oral phosphate supplementation (a total of 88 mmol sodium-potassium phosphate) [3-5], and 0.5 µg of oral calcitriol daily to enhance intestinal phosphate absorption.

**Figure 1:**
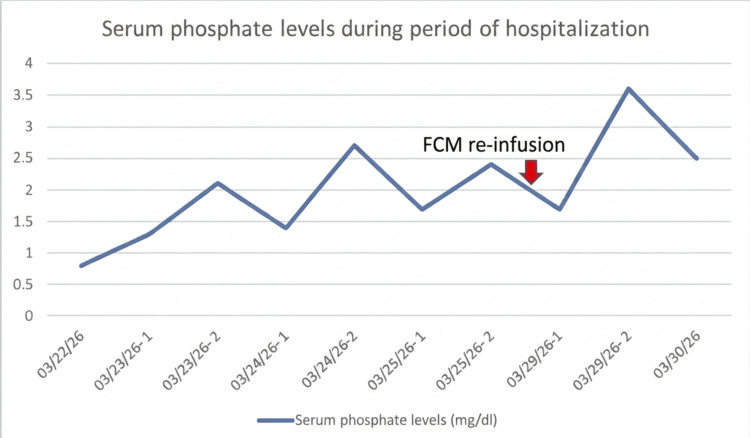
Line graph showing fluctuating levels of serum phosphate with intravenous and oral phosphate replacement.

The patient was asked to discontinue the IV iron infusion upon discharge. Serial monitoring showed gradual improvement in phosphate levels, and her neuromuscular symptoms resolved. Her discharge phosphate level was 2.7 mg/dL, and she was sent home on 0.5 µg calcitriol daily and 250 mg of sodium phosphate/potassium phosphate three times a day. Despite these phosphate replacements and strict recommendations to discontinue the iron infusion, recurrent symptomatic hypophosphatemia occurred following a single 750 mg dose of repeat iron infusion after discharge, necessitating readmission four days after discharge. It is unclear why the patient received a repeat iron infusion despite recommendations during discharge to discontinue. Her presenting phosphate level this time was 1.7 mg/dL. Her blood phosphate levels continued to fluctuate during both admissions, as demonstrated (Figure 1), despite treatment. She was ultimately discharged on oral phosphate and calcitriol, with recommendations to stop or switch FCM to other IV iron formulations and close outpatient follow-up with phosphate levels.

## Discussion

Epidemiology and risk factors

IV iron therapy is widely used in the management of iron deficiency, particularly in patients who are intolerant of or unresponsive to oral supplementation. While generally considered safe and effective, IV iron, especially FCM, has increasingly been associated with hypophosphatemia, a complication that is often underrecognized but may be clinically significant [2,6-8].

The incidence of hypophosphatemia following IV iron varies depending on the formulation used, with reported rates ranging from 20% to 50% in patients receiving FCM . Severe hypophosphatemia, although less common, can occur and may lead to clinically significant symptoms [6,7]. Randomized clinical trials have demonstrated that FCM is associated with a substantially higher risk of hypophosphatemia compared to other IV iron formulations [7].

Several risk factors have been identified, including female sex, younger age, preserved renal function, low baseline phosphate levels, and repeated or high-dose iron administration [6,9]. Patients with normal renal function are particularly susceptible due to intact phosphate excretion. Additionally, the choice of IV iron formulation plays a critical role, with FCM consistently demonstrating a higher propensity to induce hypophosphatemia compared to other preparations [7,10,11].

Pathophysiology

The primary mechanism underlying IV iron-induced hypophosphatemia involves dysregulation of FGF23, a hormone central to phosphate homeostasis. FCM increases levels of biologically active FGF23, which reduces renal phosphate reabsorption by downregulating sodium-phosphate cotransporters in the proximal tubule [6,12].

Elevated FGF23 also suppresses 1,25-dihydroxyvitamin D synthesis, resulting in decreased intestinal phosphate absorption and contributing to secondary hyperparathyroidism [6,12]. The combined effects of renal phosphate wasting, reduced intestinal absorption, and hormonal dysregulation explain both acute and prolonged hypophosphatemia. Notably, FGF23 elevations may persist for weeks after infusion, leading to sustained phosphate depletion even with supplementation [6,9].

Clinical manifestations

Patients may be asymptomatic initially, but severe hypophosphatemia (<1.0 mg/dL) can present with fatigue, lethargy, muscle weakness and myalgias. They may also experience paresthesias (perioral or extremity), tetany, seizures and cardiac arrhythmias in extreme cases [2,6,9]. Osteomalacia and pathologic fractures may also occur with chronic hypophosphatemia [6,9]. Our case exemplifies neuromuscular manifestations, with positive Chvostek’s sign, peri-oral tingling, and hand numbness correlating with critically low phosphate levels.

Differences among IV iron formulations

The risk, severity, and duration of hypophosphatemia varies significantly among IV iron formulations, as demonstrated in Table 2. FCM is associated with the highest risk due to its pronounced effect on FGF23, whereas iron isomaltoside and other formulations such as iron sucrose and ferric gluconate demonstrate a lower risk profile [7,10].

**Table 2:** Comparison of intravenous (IV) iron formulations and risk of hypophosphatemia.

IV iron formulation	Risk of hypophosphatemia	Mechanism	Notes
Ferric carboxymaltose	High	Rapid FGF23 elevation → renal phosphate wasting [6,10]	Most reported cases of severe hypophosphatemia; risk increased with repeated dosing [7]
Iron sucrose	Low to moderate [13]	Mild FGF23 increase	Usually transient, rarely symptomatic
Iron dextran	Low	Minimal FGF23 effect [6]	Rarely associated with severe hypophosphatemia; risk mostly in chronic use [6]
Iron isomaltoside	Low	Minimal FGF23 effect [10]	Generally well tolerated; safe in patients at risk [10]

These differences highlight the importance of careful formulation selection, particularly in patients with known risk factors. Current consensus recommendations advise avoiding FCM in high-risk individuals and considering alternative agents when appropriate [11].

This comparison highlights why FCM should be avoided in patients with prior hypophosphatemia or at high risk and why phosphate monitoring is particularly important after its administration.

Management

Management focuses on correcting phosphate levels and preventing recurrence. Discontinuation or avoidance of the offending agent is essential, particularly in patients with prior severe hypophosphatemia [6,11].

Phosphate repletion is indicated based on severity, with IV replacement reserved for symptomatic or severe cases and oral supplementation used for milder cases [10]. Calcitriol may be beneficial in enhancing intestinal phosphate absorption and mitigating secondary hyperparathyroidism [6,11].

Close monitoring of serum phosphate, calcium, and parathyroid hormone levels is recommended, especially following repeat IV iron administration [6,11].

Monitoring and prevention strategies

Proactive strategies to prevent severe or recurrent hypophosphatemia includes measuring serum phosphate, calcium, and vitamin D before initiating IV iron [6,11]. Rechecking of phosphate levels within one to two weeks of infusion is also recommended followed by weekly monitoring for four to six weeks in high-risk patients [6,11]. Patients with prior severe hypophosphatemia should not receive FCM; lower-risk formulations should be considered [7,11]. Patients should also be educated about symptoms of hypophosphatemia (fatigue, tingling, and cramps) and advise prompt medical attention when they experience them.

Clinical relevance

Recognition of IV iron-induced hypophosphatemia is improving, but awareness remains incomplete. Early detection may prevent complications such as osteomalacia, fractures, and neuromuscular symptoms, thereby reducing hospitalizations. Our case demonstrates that recurrence can occur despite supplementation if the offending iron formulation is re-administered, emphasizing the need for long-term follow-up and careful selection of IV iron therapy.

Limitations

A key limitation of this case report is the absence of FGF23 measurements, which would have more definitively supported the proposed mechanism underlying the hypophosphatemia. Additional concerns in the patient’s management include the use of IV iron despite the apparent lack of indication based on established criteria, as the patient may have been adequately managed with oral iron therapy.

Furthermore, a more thorough assessment of the patient’s clinical background might have identified predisposing risk factors for hypophosphatemia, thereby guiding the selection of alternative IV iron formulations with a lower associated risk of this adverse effect. Finally, the decision to administer repeat IV FCM following a prior admission for severe hypophosphatemia represents a potentially avoidable outcome, which may have been mitigated through more robust communication and comprehensive patient evaluation.

## Conclusions

Recurrent, severe hypophosphatemia is a clinically significant complication of IV iron therapy that may necessitate repeated hospitalization. Clinicians should assess baseline phosphate levels, identify at-risk individuals, and monitor phosphate longitudinally during and after IV iron administration. Discontinuation of causative formulations, targeted phosphate and vitamin D supplementation, and patient education are essential to prevent recurrence.
